# Astrocytic TGF-β1: detrimental factor in ALS

**DOI:** 10.18632/oncotarget.4786

**Published:** 2015-07-03

**Authors:** Fumito Endo, Koji Yamanaka

**Affiliations:** Department of Neuroscience and Pathobiology, Research Institute of Environmental Medicine, Nagoya University, Nagoya, Aichi, Japan

Glial cells, named after the Greek word meaning “glue”, have long been regarded just as the supporting actors in neuroscience. However, the recent research revealed their active roles in neurodegenerative diseases including amyotrophic lateral sclerosis (ALS), an adult, fatal motor neuron disease.

Using ALS model mouse overexpressing ALS-causing mutation of SOD1 (Cu/Zn superoxide dismutase) gene, we and other groups have shown that glial cells, especially astrocytes and microglia, are capable to modify the disease course of mutant SOD1 (mSOD1) mice through a non-cell autonomous mechanism [1, 2]. In a healthy condition, astrocytes play critical roles in maintenance of the central nervous system (CNS), for instance, by regulating concentration of glutamate at synapses through excitatory amino-acid transporter 2 (*EAAT2*), providing trophic supports to the neighboring neurons, and controlling synaptic functions. Earlier studies demonstrated that the focal loss of EAAT2 was observed in ALS patients as well as mSOD1 mice. Defect in clearing glutamate by ALS astrocytes supported the idea that excitotoxicity from excess glutamate was implicated in the pathomechanism of ALS. On the other hand, recent studies showed that mSOD1 astrocyte and astrocyte-like cells derived from postmortem familial and sporadic ALS patients acquired unknown toxicities selective to motor neurons *in vitro* [3]. Therefore, ALS astrocytes seem to play a role in non-cell autonomous neurodegeneration through combined mechanisms of the loss of normal functions and the gain of toxicities.

Neuroinflammation, consisted of activated astrocytes, microglia, infiltrated T cells, and the subsequent production of various inflammatory mediators, is a pathological hallmark of ALS [4] and seems to be an important component in non-cell autonomous neurodegeneration in ALS. Previous studies have shown that infiltrated T cells regulate the neuroprotective inflammatory responses mediated by microglia in SOD1^G93A^ mice [5]. In addition, our previous work has shown that mutant SOD1 expressing astrocytes accelerated disease progression in mSOD1 mice [1]. However, the neuroinflammatory factors secreted by astrocytes critical to ALS have not been elucidated.

We identified an anti-inflammatory cytokine Transforming growth factor-β1 (TGF-β1) as a new determinant of ALS disease progression. TGF-β1 has key roles in immune homeostasis and tissue injury and is elevated in the blood and cerebrospinal fluid (CSF) of the patients with ALS. In our recent study, we found that TGF-β1 was upregulated in the spinal cord astrocytes of sporadic ALS patients and symptomatic mSOD1 mice. To investigate the role of astrocytic TGF-β1, we crossbred SOD1^G93A^ mice with the transgenic mice overproducing TGF-β1 specifically in astrocytes [6]. Contrary to our initial expectation that overexpression of TGF-β1 might prolong survival times of SOD1^G93A^ mice through an anti-inflammatory action of TGF-β1, we surprisingly found that the double transgenic mice died about 10 days earlier than SOD1^G93A^ mice without any difference in the age of onset, indicating that astrocytic TGF-β1 accelerated the disease progression of mSOD1 mice. How does astrocytic TGF-β1 accelerate disease? We found that astrocytic overproduction of TGF-β1 in mSOD1 mice resulted in lower production of the neurotrophic factor, insulin-like growth factor-I (IGF-I) in deactivated microglia and fewer infiltrating T cells with an IFN-γ-dominant milieu: astrocytic TGF-β1 dampened neuroprotective inflammatory reaction by microglia and T cells. In contrast, we found that selective deletion of mSOD1 in astrocytes slowed disease progression of SOD1^G37R^ mice [1] with a lower level of TGF-β1 in astrocytes. These results indicated that astrocytic TGF-β1 negatively regulates disease progression of mSOD1 mice. To translate these findings into the experimental therapy, we have demonstrated that pharmacological administration of TGF-β signaling inhibitor after disease onset modestly but significantly extended survival time of SOD1^G93A^ mice [7].

In summary, astrocytic TGF-β1 is a detrimental factor to dampen the beneficial neuroinflammation in ALS. Moreover, TGF-β1 is also implicated in the other neurodegenerative diseases such as Alzheimer's disease. Our finding extends the notion that neuroinflammation is not always detrimental but actually has both the beneficial and detrimental forms. It becomes a critical question in neurodegenerative diseases how to preserve the beneficial portion of neuroinflammation and dampen the toxic one to develop a novel therapy for neurodegenerative diseases.
